# A Metric for Evaluating a Transformation of Subscription-Based Journals into Open-Access Journals

**DOI:** 10.1007/s10916-020-01651-1

**Published:** 2020-10-06

**Authors:** Corinna Mielke, Stefanie Kuballa, Mareike Schulze, Reinhold Haux

**Affiliations:** 1 Peter L. Reichertz Institute for Medical Informatics of TU Braunschweig and Hannover Medical School, Mühlenpfordtstr. 23, 38106 Braunschweig, Germany; 2 Peter L. Reichertz Institute for Medical Informatics of TU Braunschweig and Hannover Medical School, Hannover, Germany

**Keywords:** Open access, Evaluation metric, Transformation, Medical informatics

## Abstract

Open Access is an upcoming paradigm to communicate scientific knowledge. The Trans-O-MIM Project works on strategies, models, and evaluation metrics for the goal-oriented, stepwise, sustainable, and fair transformation of established subscription-based scientific journals into open-access-based journals. This research intends to present an evaluation metric and the associated identified appropriate parameters for such transformations. To develop the evaluation metric, it has been implemented in the context of a business management method for planning, steering and controlling action and corporate strategies. The central element was a 3-step procedure for developing the metric. In stage 1 necessary preconditions for a transformation were considered. Stage 2 is the actual elaboration of the evaluation metric by means of a scenario analysis and stage 3 comprises the exemplary testing at the journal Methods of Information in Medicine. The three methodological steps have primarily resulted in 5 scenarios with 9 different final states from the scenario analysis. Thus, the metric is now composed of these 5 scenarios, which can be used to evaluate the success or failure of a transformation. A list of 65 suitable parameters to measure changes in scenario were compiled. So, it is possible to evaluate the transformation and to find the current final state. Parameters like submissions, publications, and time as well as the scenario states could be applied to the transformation process of the Methods of Information in Medicine journal. The proposed evaluation metric can be used to evaluate the transformation processes of subscription-based journals into open-access-based journals.

**Electronic supplementary material:**

The online version of this article (10.1007/s10916-020-01651-1) contains supplementary material, which is available to authorized users.

## Introduction

Open Access (OA) is an important and established form of knowledge and science communication [1]. The review by Kuballa shows that in the field of medical informatics there are various variants for publication in Open Access and that researchers in this field have a free choice between these variants [2]. However, the central challenges identified were the uncertainty of authors with regard to the different publication options, the financial framework conditions and the reluctance of journals to promote open access publications [2]. Therefore, OA is advancing and increasingly complementing the traditional form of scientific communication.

To investigate the possibility of switching under fair conditions from the existing science communication model to science communication with OA, the project ‘Strategies, models and evaluation metrics for the goal-oriented, stepwise, sustainable and fair transformation of established subscription-based scientific journals into open-access-based journals with Methods of Information in Medicine as example’ (Trans-O-MIM) – funded by the German Research Foundation (DFG) – was carried out. Haux et al. describe the details and general conditions of this project in their 2016 paper [3].

This project aims to transform an existing Subscription-based Journal (SJ) into an Open Access-based Journal (OAJ) under fair conditions [3]. Haux et al. present a tandem model to enable gold OA publishing [3]. In this way, a SJ is gradually transformed into an OAJ with gold OA. Haux et al. use the SJ Methods of Information in Medicine (MIM) as an example of this transformation [3]. The project also required the transformation process to be monitored [3]. Thus, the authors set the goal of developing a metric with which it is possible to observe, monitor, and evaluate the transformation of a SJ into an OAJ.

The current status of the evaluation of OAJ refers to testing and evaluating Journals and the articles they contain [4]. An evaluation metric for the evaluation of a transformation process from one type of journal to another or a comparable approach could not be wound in the literature. However, there are guidelines that can be used to create and build evaluation metrics, especially in the field of open science [5, 6]. Thus, it was possible to create evaluation metric for the concrete scope of the transformation of a SJ into an OAJ according to the specifications in the project proposal.

This paper reports on this metric. The tool of choice for the metric is a scenario analysis, which is used in the context of futurology e.g. as a method for the observation and evaluation of processes in economics [7, 8]. The goal of scenario analysis is to accompany and evaluate the transformation process of a subscription journal (e.g., MIM) into an OAJ with Gold Standard (e.g., Methods Open). With help of the accompanying evaluation, decisive parameters for success or failure of the transformation can be observed and evaluated over the duration of the changeover. This information is helpful to guide the process of transformation and possibly to promote it through targeted measures such as targeted marketing or possibly financial incentives. To steer the outcome of the transformation in the desired direction. The developed scenarios should therefore serve as indicators for steering and influencing the transformation.

For this purpose, the parameters, influencing and disruptive factors that influence the transformation process are determined. These factors are then used to create scenarios to describe the possible final states after a transformation. In this way, assessments can be made to predict the result of the transformation process. With regard to the objectives, the concrete area of scenario analysis lies in the monitoring and evaluation of the transformation from MIM to Methods Open. The tandem model is used as a basis, whereby the journal is divided into a subscription part and an OA part. Both parts are compared and evaluated. Thus, data are collected and evaluated on parameters that are decisive for the success or failure of the transformation. The result is compared with the defined scenarios and the trend in the transformation is determined based on the current figures. Thus, it is examined whether conversion from a subscription journal to an OAJ is possible using the tandem model.

The scenario analysis consists of three development phases: the preparation phase, the scenario development phase, and the monitoring or evaluation phase. The methods and results of these three phases are described in the following sections. The preparation phase for the transformation is an important and necessary step, especially on the part of the publisher, as it allows the possible framework conditions and tasks, but also the first environmental and influencing factors that are decisive for the scenario analysis to be determined. In addition, these tasks are important for the publisher, so that it can be assessed whether a transformation is at all possible for the publisher. The second phase is based on the results and insights from phase one and now enables the exact determination of the decisive parameters, influencing and disruptive factors for the entire transformation process as well as the scenarios resulting from the possible developments of these parameters. The third phase then uses the example of MIM to show an implementation of the transformation with accompanying evaluation and assessment of the current state of the journal through the defined scenarios and the current figures for the decisive parameters for success and failure of the transformation.

## Preparation Phase for the Transformation of a SJ into an OAJ

Due to the lengthy process of transforming a SJ into an OAJ, a publication service provider is confronted with various questions and necessary preparation work. During the Trans-O-MIM project, we were able to identify the following five essential and necessary preliminary tasks for publication service providers (PSP).

For the **journal-specific definition of OA,** it must be decided how and where future articles are to be published. For example, whether articles are to be published on the PSP’s website or full-text indexing within a database such as PubMed Central. Deciding on the appropriate license is also an important factor.

An important point in the preparation phase is to establish the **authors’ interest** in an OAJ and willingness to publish. A good instrument for investigating is a survey and continuous communication with stakeholders, either through social media or being present at congresses and conferences.

Since subscription fees are not charged for an OAJ, alternative **financing** methods must be considered. These could, for example, take the form of advertising revenue, subsidies from professional associations, or an “author pays” model in the form of the so-called Article Publication Charges (APC) [9]. To be able to assess the feasibility of an OA transformation and the amount of the APCs, a cost statement is a central component of the preparation phase. It is important to be conscious of the advantages to orientate oneself to the current funding ceilings of research funding agencies and to implement a model for authors from developing countries. With regard to financial feasibility, planning is just as important as dealing with a decline in submissions. A transformation carries the risk that authors will be more reluctant to submit articles. This means that a financial collapse could see compensation from reserves or other funds for a certain period of time.

The effort required to **convert the editorial processes** in the run-up to an OA transformation should not be underestimated. Editing and publication of OA articles require the creation of a suitable workflow. The article layout and the integration of clean and complete metadata must be defined. Also, the processes related to invoicing, such as the rapid dispatch of invoices and cross-departmental information on incoming payments, must be worked out. Functioning communication is also important for final publication of the articles. For this purpose, the editorial office, accounting department, and the electronic media department must be integrated into the communication process to be able to place finished and paid articles online.

The preparation of a **marketing strategy** is also part of the preparation phase of a transformation. Here the aim is to inform all stakeholders early and continuously about the changes in the journal. Thus, professional societies should be actively informed and involved in the project. Of particular importance are the authors. They must be informed about the innovations and convinced of the advantages. To ensure sustainability, the marketing strategy should be long-term and planned at least one year in advance.

## Scenario Analysis for the Transformation Process of a SJ into an OAJ

To evaluate the transformation, a scenario analysis is recommended for the preparation phase, in which the possible final scenarios of a journal transformation are developed. It is then possible to use these scenarios to make an assessment in the monitoring phase. Scenario analysis is a method of strategic control in which alternative environmental scenarios are created. A scenario represents a possible future situation, from which different conclusions and development possibilities can be derived [10].

### Procedure for a Scenario Analysis

In every scenario development, the starting point for the development of a scenario is the current status, i.e. the actual situation at time t_0_. Depending on the development of the influencing factors and the environment, different scenarios can be described for different points in time in the future [11]. The scenario is time-dependent since future situations are described. The further into the future the scenario is, the more uncertain the underlying data becomes, and the more variations of the scenario are possible [10, 12]. It is neither necessary nor desirable to consider all possible scenarios. A reasonable limitation to a few, meaningful, as different and stable scenarios as possible is sufficient to cover all directions of development in an exemplary manner and to be able to represent the scenario space completely. A reasonable number should result from the concrete problem and be as small as possible [11]. To limit the complexity of the scenario analysis, the minimum requirement is that at least the following three types of scenarios are created [10, 12].

1. Best-case scenario – describes the most positive case for the transformation to be performed.
2. Worst-case scenario – describes the worst case for the execution of the transformation.
3. Trend scenario – should occur if the trend of the underlying data and parameters remains the same (also known as the business-as-usual scenario).

Thus, the definition of scenario analysis as “a systematic methodology for developing scenarios” [13] or “a system of different components with the aim of developing alternative scenarios from a given problem” [14] is not a good one. For the elaboration of scenarios, scenario analysis follows a fixed process character with the analysis phase, the forecast phase and the synthesis phase. “The phases are based on each other and are run through one after the other” [11]. In the analysis phase, “the precise delimitation and definition of the problem” [11], “the compilation of all basic information required to characterize the initial situation” [11] and “the development of all important areas of influence” [11] are carried out. In the prognosis phase, the central tasks are “the establishment of reasonable, coherent, future developments of the areas of influence” [11] and “the examination of their stability with the help of disturbance events” [11]. The third phase then contains “the final scenario formulation for the actual problem” [11] as well as “first thoughts of implementation” [11] are possible. For the individual elaboration of the tasks in these three phases, there are many different fine divisions in literature [15–20]. The standard time period considered for a scenario analysis is between five and ten years [10].

### Procedure of a Scenario Analysis for the Transformation Process of a Subscription-Based Journal into and Open-Access Based Journal Using the Example of the Journal Methods of Information in Medicine

The system boundaries, basic conditions, participants, target groups, parameters, and environmental influences were developed in cooperation with Schattauer GmbH (Schattauer). This meant the processes of the publishing house for publishing science journals could be analyzed. By interviewing experts during project meetings, we received relevant information on the respective partial aspects of the scenario analysis and were able to derive the described scenarios on this basis. The inclusion and analysis of the scientific journal MIM was decisive in this context.

#### Target Setting

##### Defining System Boundaries and Basic Conditions

In our opinion, the transformation of a SJ should meet the requirements of being goal-oriented, stepwise, sustainable, and fair [3]. These requirements serve as basic conditions for the scenario analysis. As many of these requirements as possible should be considered during the transformation.

Parallel to these requirements is another category of basic conditions for scenario analysis. These are financial conditions. In particular, the initial investment costs and running costs play a central role. The basic conditions that must be taken into account for the running costs are:

- The cost coverage should be available
- The business model should cover production costs
- Financial resources must be made available

For investment costs, the basic condition to be taken into account is:

- One-off internal costs for the transformation

##### Identifying Participants and Target Groups

Individuals in the group of participants describe the roles they must participate in during the transformation process and the decisions they must make. The following participants are identified as being relevant groups: executive management, accounting and finance department, editorial offices, marketing department, press department, electronic media department, production department, advertising department, and publishers.

The target group describes the groups that are addressed by the transformation process. The groups’ publication organizations (publishers and professional societies), authorship, readership, and advertising clients are identified as target groups for the transformation.

##### Defining the Parameters and Control Parameters for Scenario Analysis

For creation of scenarios, identification of parameters influencing journal and transformation process is an essential step. These parameters are an essential part of the scenarios, and their specific developments results in the current valid trend scenario. Definition of characteristics of the parameters is necessary for statements and measurability of the respective scenarios. The parameters are divided into primary parameters and secondary parameters (as listed in Table 1). The primary parameters include the categories of publication parameters and financial parameters. The development of these parameters directly and immediately influences the scenarios that occur. The secondary parameters are parameters relating to process quality, impact, reach, dissemination, compliance, scientific indicators, and other parameters relating to the author and customer perspectives. They provide information on the current quality and reputation or dissemination and future developments in these areas. Their observation can help to identify and prove possible causes for the development of primary parameters. The recording of the secondary parameters should therefore serve as a safeguard and control for the recording of the primary parameters.

**Table 1:** Overview of the defined primary and secondary parameters for the scenario analysis, sorted into categories

**Primary parameters**	**Secondary parameters**
***Category: publication parameters***	***Category: quality parameters***
• Number of publications in the subscription part • Number of publications in the OA part • Difference of publications in both journal parts (difference between the number of publications in the subscription part and the number of publications in the OA part) • Number of submissions to the subscription part • Number of submissions to the OA part • Difference of submissions in both journal parts (difference between the number of submissions in the subscription part and the number of submissions in the OA part) • Acceptance rate of the subscription part • Acceptance rate of the OA part • Difference between the acceptance rate of the subscription part and the OA part • Number of citations for the subscription part compared with the number of citations for the OA part • Access rate (e.g., download rate) for the publications ***Category: financial parameters*** • Cost/ revenue - Revenue from the subscription part - Revenue from the OA part - Revenue from promotions (advertising revenues) Advertising revenues OA part Advertising revenues subscription part - Operating costs for the subscription part and the OA part Variable costs/ running costs Fixed costs Development costs (cost for the transformation)	• Quality of publications - Relative Citation Ratio [21] Citation rate of one publication compared with the citation rate of the research field - Value of publications • Time aspects - Duration of the processing procedure Needed process time by the publisher Duration from acceptance to release of the publication taking Online First into account for the subscription part • Author satisfaction • Quality of process of the subscription part versus the OA part - Simplification of process - Speed-up of process - Breakdown and comparison of workflows from the subscription part and the OA part ***Category: other parameters*** • Accessibility - Number of individuals who have access to the journal - Usage statistic of online segment through the number of downloads and DOI access • Supply - To which individuals can the journal be offered? • Demand - Which individuals benefit from the journal? • Comparison between the user group of the subscription part and the user group of the OA part - Comparison of age - Comparison of origin • Comparison of the topics from the subscription part and the OA part

Table abbreviations: OA – open access.

#### Environment Analysis

The environment analysis includes a determination of external areas of influence with associated influencing factors, as well as identification of possible disturbance variables. The external areas of influence for a journal transformation are diverse. Possible spheres of influence are legal decisions or changes in the legal situation, economic influences due to changes in funding and financing, social influences due to the interests of authors or readers, and technological influences due to rapid technological developments. Impacts on the successful transformation of a SJ into an OAJ can be assumed from the status of the journal in the scientific societies, the decisions of funding organizations regarding funding OA articles, access to funding resources, decisions by state governments, business decisions by publishers, trends in OA publications among authors and state governments, and developments in the journal’s impact factor. With regard to the influencing factors associated with these areas of influence, it is only realistic to consider factors that have a probability of occurrence and evaluate their impact on the transformation. Examination of the spheres of influence shows that determining the probability of occurrence and their impact on the project is not possible because certain probabilities and frequencies are not available or cannot be determined. Two central aspects could be identified for the internal and external influencing factors. On the one hand, this is the increase in internal costs due to a high number of submissions, which only leads to a small number of publications. On the other hand, the introduction of a fee for the use of Pubmed-Central’s services must be considered as an external disturbance variable. Both disturbance variables have an impact on the costs of the transformation.

#### Scenario Creation

**Fig. 1:**
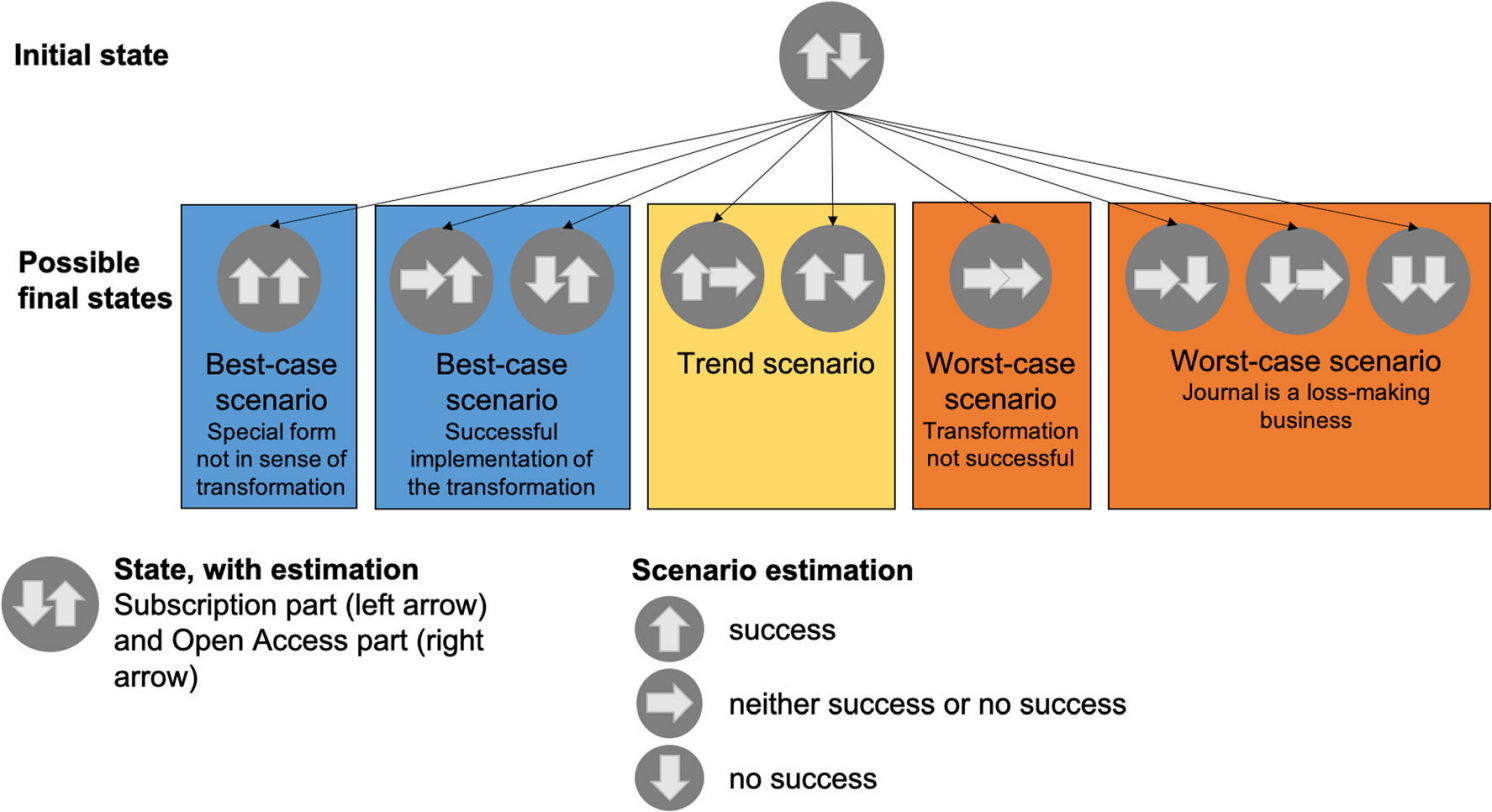
Process of scenario analysis from initial status to possible endpoints and places them into the possible scenario groups

The scenario analysis starts from the initial state and then presents several possible final states for the outcome of the transformation. The possible states are described with an evaluation of the subscription part and the OA part. For the scenario analysis, three evaluations can be identified for each journal part. These are a positive evaluation, neutral evaluation, and negative evaluation in the sense of transformation. With regard to these assessments, there are nine possible variants for the final states. These nine possible final states of the transformation can be summarized into five scenarios. This is possible because some of the final states have the same effect on the journal when considering the parameters that determine the success or failure of the transformation. The division of the final states into scenarios is shown in Fig. 1. This shows, for example, that it does not matter for the successful transformation whether the share of publications in the SJ part decreases or remains the same as long as the number of publications in the OA part of the journal increases. In this case the development in terms of the transformation is positive and can therefore be assigned to the best-case scenario. Only if the number of publications in the SJ part continues to increase, another scenario results, which can also be considered a best-case scenario but is not in the sense of the transformation of the journal. These mergers for the remaining final states. The resulting five scenarios are divided into two worst-case scenarios, two best-case scenarios, and one trend scenario. The first worst-case scenario describes a situation for the journal that is possible because of the transformation but is not foreseen by the publisher. The second worst-case scenario describes a situation in which both parts of the journal are affected by the transformation in such a way that, from a business perspective, the maintenance of the journal is no longer viable. The trend scenario describes the state that the transformation does not result in any changes to the initial state of the journal. The continuation of the journal as a SJ would still be possible after this scenario. The first best-case scenario describes the successful transformation from a SJ to an OAJ. The second best-case scenario describes the situation in which the number of submissions to both journals parts increases alongside two separate journals, each of which can exist independently. The following detailed description of the five possible scenarios show the primary parameters to be considered. The selected primary parameters have an immediate effect on the two journal sections and the development of the possible final state of the scenario analysis.

#### Worst-Case Scenarios

**Case one – Transformation not successful:** This describes the state that the transformation could not be successfully implemented. The decisive parameters here are a low number of submissions to the OAJ section, which then leads to a low number of publications. This has a significant impact on the parameters’ costs and revenues, which can lead to a situation where costs can be covered but profits are few or non-existent. Thus, the continued existence of the OAJ section is dependent on the SJ section. However, if the number of submissions for the SJ part is reduced, this may result in costs being covered but no profit made from this journal part. This means the overall situation becomes non-viable from a business viewpoint. The consequential measure from such a scenario would be to discontinue the journal completely. The central parameters that influence this scenario are:

- Low willingness to send in submissions
- Small number of publications
- Quality of submissions does not meet the requirements of the review process
- No income
- No cost recovery

**Case two – Journal is a loss-making business:** This describes the case that both parts of the journal no longer cover costs due to the transformation. Therefore, the entire journal is to be considered a loss-making business. In this case, the measures for the publisher are merely discontinuation of the journal. The factors that significantly influence this scenario are:

- Both journal sections receive too few submissions
- Both journal sections are no longer sustainable due to their respective business models
- Subscriptions are in sharp decline
- Regularity of article publications
- Influence on submissions

#### Trend Scenario

This scenario describes the development of the two journal sections if there are no significant changes in the initial publication figures and costs. Both journal sections remain unchanged, but the transformation to a pure OAJ is not successful. However, the subscription section is still stable and can be continued as an independent journal. The essential factors for observing the achievement of these scenarios are:

- Publication rate in OA section
- Publication rate in the subscription section
- Cost recovery in the OA part
- Cost recovery in the subscription part

#### Best-Case Scenarios

**Case one – Successful implementation of the transformation:** Case 1 describes the successful implementation of the transformation from SJ to an OAJ. A successful transformation is given if the number of publications in the OA part of the journal increases steadily, and the number of publications in the subscription part decreases steadily. At the same time, a positive cost development with profit generation for the OA part of the journal must be achieved. The costs for the subscription part of the journal should slowly decrease, but there should not be negative cost recovery. The main factors and characteristics for monitoring the achievement of these scenarios are:

- Publication rate in the OA part is higher than in the subscription part
- Publication rate in the subscription section gradually reduces
- OA part grows bigger and bigger
- Subscription part can be completely transferred to the OA part

**Case two – Special form not in the sense of transformation:** Case 2 describes a special form that can result from the transformation. This special form includes the possibility that both parts of the journal continue to show positive development in publication figures and cost recovery. It should be noted, however, that this special form is not intended to be a transformation. This case can occur if the publication rate in both journal sections shows continuous improvement. In addition, a positive cost recovery with profit generation in both parts of the journal would be necessary to achieve this scenario. The result of this special form would be that the attempt of transformation has contributed to the fact that both journal sections can continue to exist as independent journals. The main factors and their development possibilities to observe the achievement of these scenarios are:

- Publication rate in OA section
- Publication rate in the subscription section
- Cost recovery in the OA part
- Cost recovery in the subscription part

The metrics result from the scenarios developed in the scenario analysis and the defined parameters. The influence of the parameters is an essential point, which allows the development of the desired scenario to be controlled. Not all parameters are of equal importance. Thus, the primary parameters have a direct influence on the development of the scenarios and thus on the development of the transformation. Whereas the secondary parameters have more of an overall evaluation character for the journal. The direct influence on the transformation and thus also on the scenarios is rather small. They can therefore be seen as additional information to assess the scientific character and acceptance of the journal in the scientific community over several years, e.g. to help sharpen the target group or to investigate measures for scientific influence.

## Applying the Evaluation Metric for the Monitoring Phase of the Transformation of MIM

For the evaluation of the transformation process, the primary parameters from the category publication parameters and the time-related secondary parameters from the category process quality parameters should be considered. The selection of the parameters was based on the criteria to which were subjected within the framework of the Trans-O-MIM project and on the requirements of the publishers Schattauer und Georg Thieme Verlag KG (Thieme) for the transformation of MIM. Predominantly these were the easy or even public availability of the used data as well as the simple realization of possible calculations and evaluations. The remaining parameters are just as relevant for the evaluation of such a transformation, but they are better applicable in the context of publisher-internal evaluations. A data set based on information and data from Manuscript Central™ [22], the MIM webpage (status February 2020) [23], and PubMed.gov [24] was compiled for the period January 2014 to September 2019. For the analysis we decided to focus on the data of PubMed.gov as these data could be used best. The database includes all original articles and all Focus Theme original articles published in the same period. Publications that did not correspond to this classification, such as editorials and Focus Theme editorials, discussions, and invited contributions from the editor, memorandums, white papers, letters to the editor, addendums and errata, were removed from the data set. For the subscription part, we use the online first date as the publication date (if applicable). The remaining data set has been divided for evaluation of the primary and secondary parameters. For evaluation of the primary parameters, a direct comparison was made between the subscription part and the OA part of MIM. This includes all publications and submissions of the two journal sections as well as the resulting acceptance rates since the introduction of the OA part in January 2017. For evaluation of the secondary parameters, a comparison was made between the two journal sections since January 2017, as well as a comparison of the time factors before and after the transformation with data from January 2014 onwards. For the before-and-after comparison, the cut-off date of 1 January 2017 (introduction of the OA part of the MIM) was established. A compilation of all the figures can be found in Online Resource 1.

### Publication Parameters: Submissions, Publications and Acceptance Rate

The presented data report the current status as a partial result of the scenario analysis after two and a half years after transformation.

**Fig. 2:**
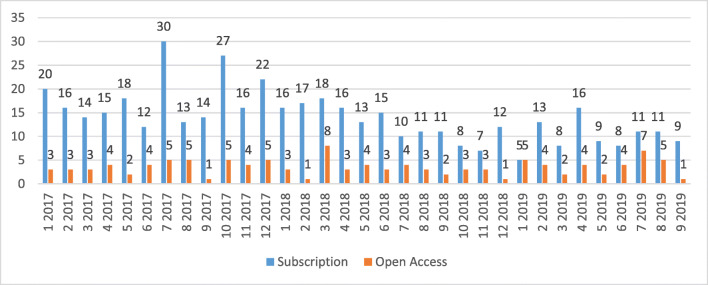
Number of submissions to the MIM journal in the period January 2017 to September 2019, divided into submissions for the subscription part and the Open Access part of the journal

The numbers of this dataset show that submissions in the subscription part of the journal decreased in the last two and a half years. Whereas figures for the OA part show as almost constant over the entire period. The concrete figures for the individual months from January 2017 to September 2019 are shown in Fig. 2.

**Fig. 3:**
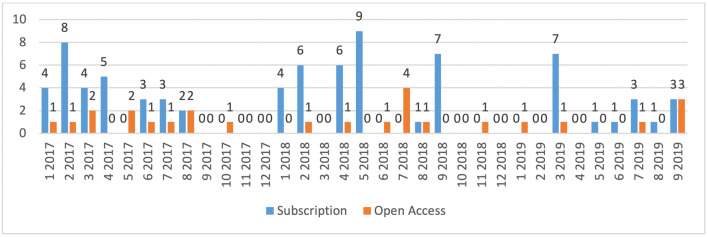
Number of publications in the MIM journal in the period January 2017 to September 2019, subdivided according to submissions for the subscription part and the Open Access part of the journal

A comparison of the figures for publications in MIM also shows a consistently low trend for publications in the OA part and a strongly fluctuating trend in figures for the subscription part. The total numbers for the period January 2017 to September 2019 are shown in Fig. 3.

Tables 2 and 3 show the figures for submissions and publications for the respective journal sections. We have scaled the figures for 2019 using the factor 12/9 to ensure comparability between years because for 2019, we only have data from January to September. We have also assigned the development of these figures to the scenarios that occurred in each year. This shows the change from the initial boom of the journal to a slump in submissions and publications for the subscription part and rather constant figures for the OA part.

**Table 2:**
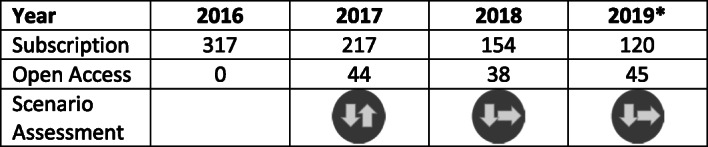
Number of submissions per year divided by the subscription and Open Access parts (*with adjusted numbers for 2019) and the scenario assessment according to Fig. 1, with the left arrow for the subscription part and the right arrow for the Open Access part

**Table 3:**
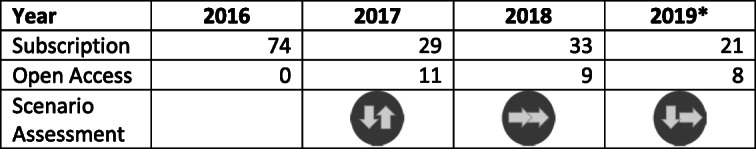
Number of publications per year divided by the subscription and Open Access parts (*with adjusted numbers for 2019)) and the scenario assessment according to Fig. 1, with the left arrow for the subscription part and the right arrow for the Open Access part

**Fig. 4:**
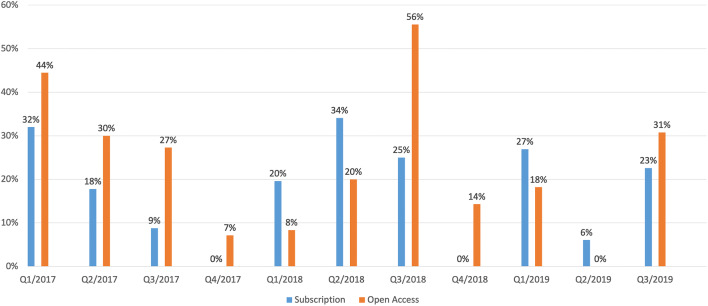
Acceptance rate per quarter for the MIM journal over the period of January 2017 to September 2019

Based on these numbers, we have calculated the quarterly figures for submissions and publications. From these figures, we were able to determine the acceptance rate for the quarters Q1/2017 to Q3/2019. The total figures can be seen in Table 4. The graphic representation in Fig. 4 shows that the acceptance rate for the OA part is slightly higher than for the subscription part. However, the reviewers do not know whether an article was submitted to the OA part or the subscription part. Possible reasons for this could be the quality of the submitted contributions or the financing of them, for example, through research funding, which is then subject to different scientific conditions.

**Table 4:** Absolute figures of submissions and publications as well as acceptance rates per quarter in the period January 2017 to September 2019 for the MIM, divided into the subscription and Open Access parts

	Subscription			Open Access		
	Publications	Submissions	Acceptance Rate	Publications	Submissions	Acceptance Rate
Q1/2017	16	50	32%	4	9	44%
Q2/2017	8	45	18%	3	10	30%
Q3/2017	5	57	9%	3	11	27%
Q4/2017	0	65	0%	1	14	7%
Q1/2018	10	51	20%	1	12	8%
Q2/2018	15	44	34%	2	10	20%
Q3/2018	8	32	25%	5	9	56%
Q4/2018	0	27	0%	1	7	14%
Q1/2019	7	26	27%	2	11	18%
Q2/2019	2	33	6%	0	10	0%
Q3/2019	7	31	23%	4	13	31%

### Process Quality Parameters: Time Factors

**Fig. 5:**
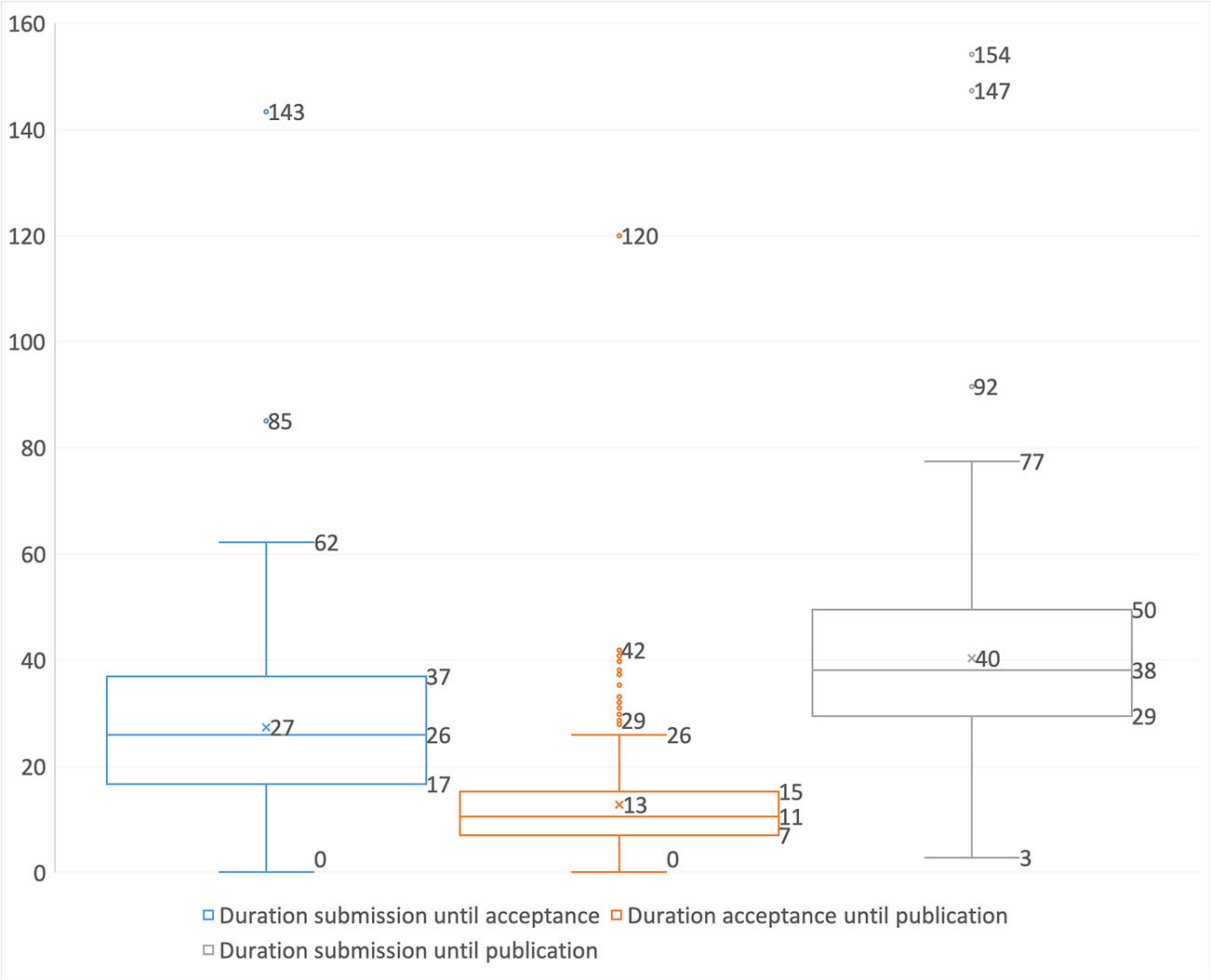
Box plots for the duration of the manuscripts, from submission to acceptance to publication, for the entire period 2014 to 2019 for all manuscripts (indicated in weeks with whiskers of length 1,5 × IQR, □ Q75 - Q25, median, o - outlier and x - mean)

For analysis of the transformation, we also looked at the secondary parameter time factor: analyzing the time between important steps of the publication process. To do this, we looked at a larger time period to compare the submissions and publications for both journal parts. The time period for analyzing the time factor starts in January 2014 and ends in September 2019. The distribution of all data are shown in Fig. 5 and are divided by different time factors. From this presentation, it can be seen that the entire publication process takes an average of about 40 weeks for a manuscript to be published. These 40 weeks are divided into 27 weeks for the review process and 13 weeks for the publisher’s editing process. Over the entire six-year observation period, there were two extreme outliers, one of which had a long review process and the other, which had a lengthy editing process by the publisher. The following evaluations are intended to present the more precise details for before and after the transformation, as well as for the two different journal parts.

### Comparison before and after Transformation

**Fig. 6:**
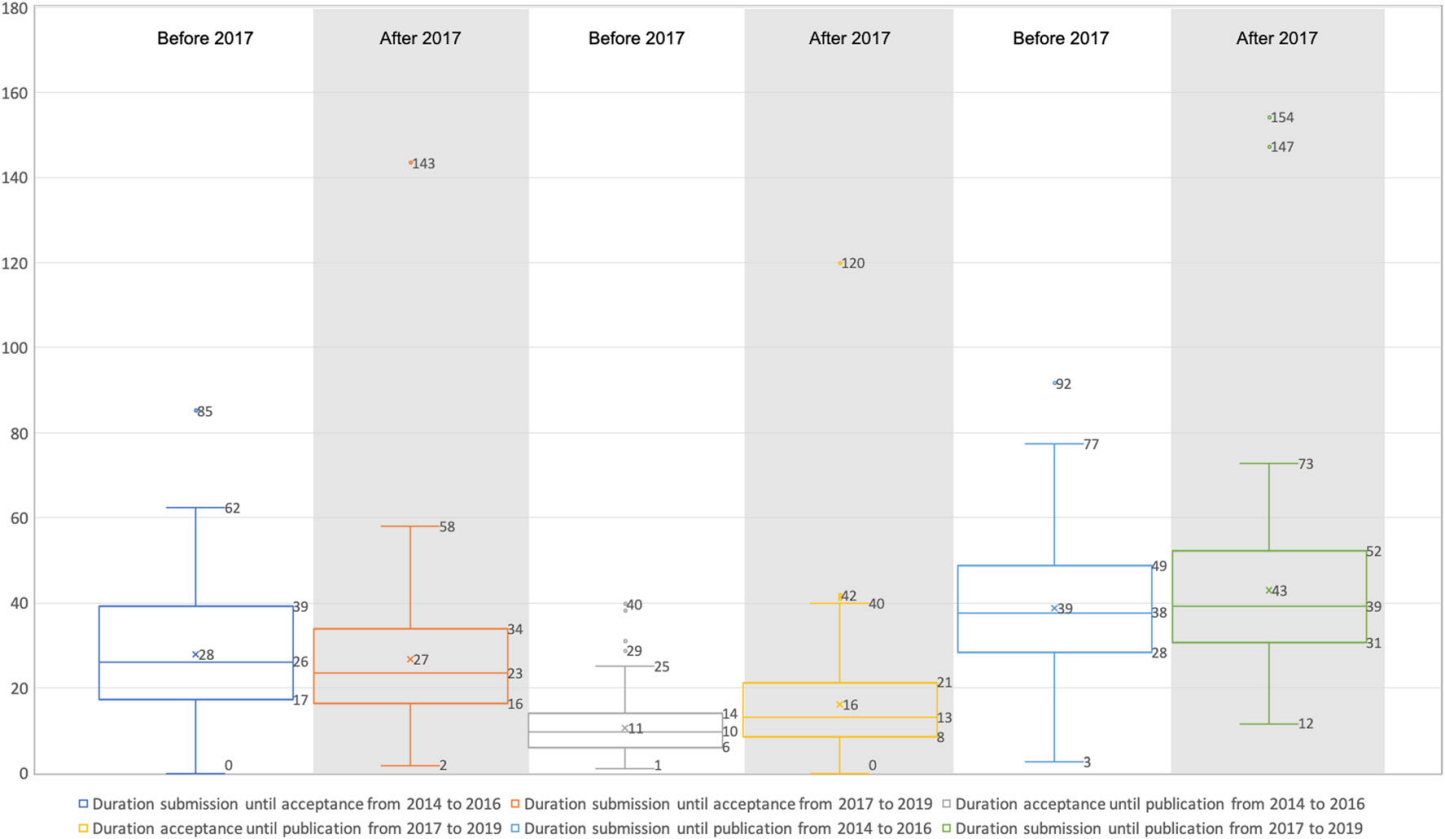
Box plots for the duration of the manuscripts from submission to acceptance to publication, for the periods before (2014 to 2016) and after (2017 to 2019) MIM’s transformation (indicated in weeks with whiskers of length 1,5 × IQR, □ Q75 - Q25, median, o - outlier and x - mean)

First, we looked at the time factors before and after transformation. The box plot in Fig. 6 shows that the differences between the time periods before and after transformation are not high for the whole publication process, with the duration from submission to acceptance being almost the same. For the publication duration process (from acceptance to publication), the figure shows that the workflow after the transformation requires more time, resulting in a longer time period of around 5 weeks to publish a manuscript after its acceptance. This could mean that the transformation had zero impact on the review process but did have an impact on the publishing workflow. Another impact could be the change in publishing houses in 2018. This means that the extra 5 weeks could be a result of the different workflows between the two publishing houses. Another striking feature is that extreme outliers also occur after MIM’s transformation in 2017. A reason for this could be our small number of 300 publications over the six-year period. For the whole time, these outliers show that there were only two manuscripts having such a long processing time. For one manuscript, the review phase was considered extremely long and could be due to several different reasons. For the other manuscript, the publication workflow was extremely high and the reason for these delays not being known.

### Comparison between the Subscription and Open Access Parts of MIM

**Fig. 7:**
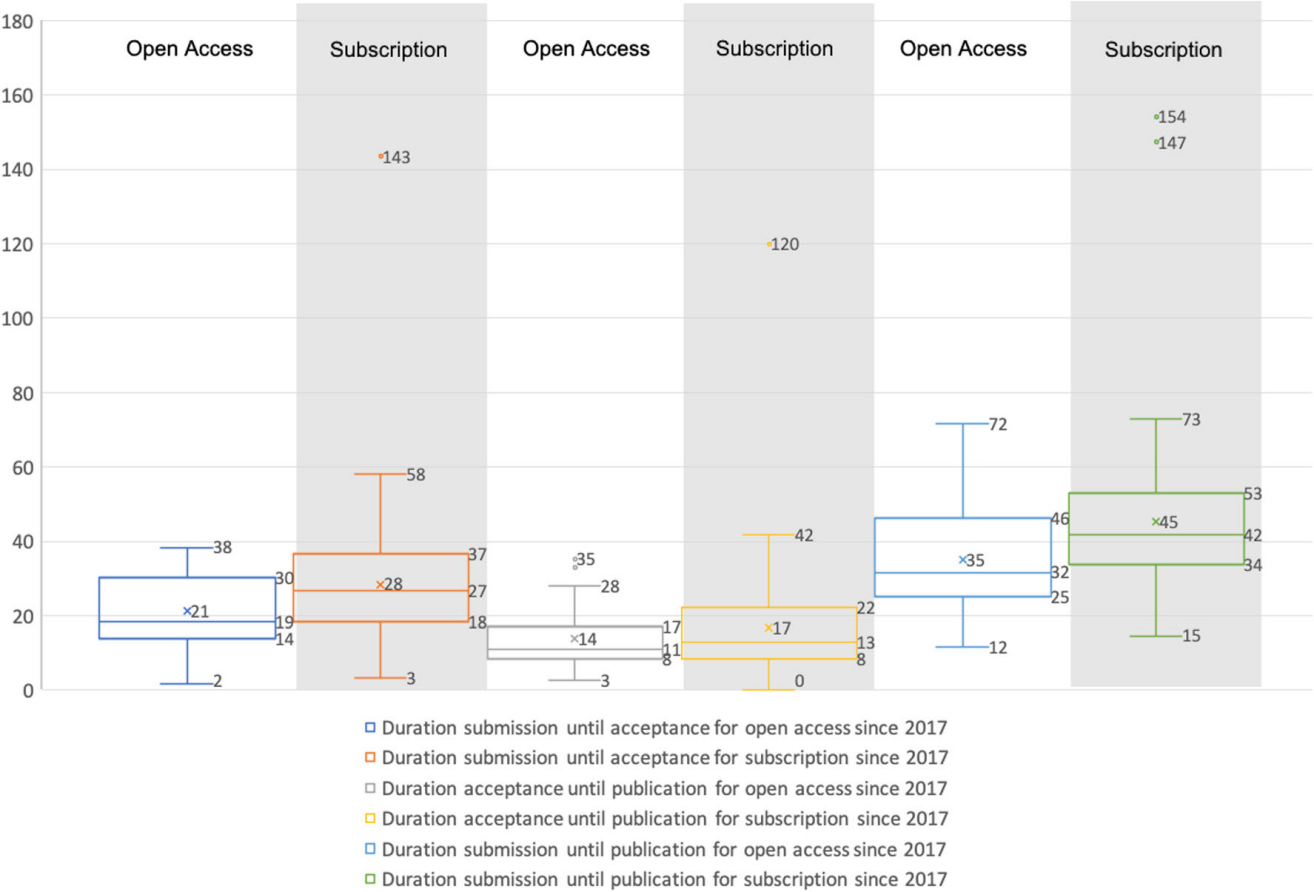
Box plots for the duration of the manuscripts from submission to acceptance to publication, divided into subscription and Open Access parts for the period January 2017 to September 2019 (indicated in weeks with whiskers of length 1,5 × IQR, □ Q75 - Q25, median, o - outlier and x - mean)

A comparison of the two journal sections of MIM shows that the publication process for a manuscript in the subscription section takes an average of about 10 weeks more than for a manuscript in the OA section (see Fig. 7). This long duration is due to the fact that both the review process and the editorial process for the subscription part have higher durations. On average, the review process takes about 7 weeks longer, and the editorial process about 3 weeks longer. This gives OA articles a significant time advantage over subscription articles. However, the reasons behind this advantage, especially during the review process, are not known. Especially since the reviewers do not receive information about whether an article is from the OA part or the subscription part of the journal. The three weeks difference for the editorial process could be an effect of the small number of publications considered in total and thus not be meaningful in relation to the transformation. In addition, this evaluation shows that the two outliers occurred in the subscription part, which distorts the mean value upwards. If we look at the median, we can see a significant difference of 8 weeks indicated for the review process, but for the editorial process, the difference in duration from acceptance to publication approaches two weeks. The influence of the transformation on these processes is, therefore, not derivable and recognizable.

After considering the individual parameters in comparison to each other as well as before and after the transformation, an overall evaluation of the new state can now be derived for the MIM on the basis of the resulted numbers and in accordance with the rules of a scenario analysis. Based on the current numbers, the transformation of MIM should be assigned to a worst-case scenario. In particular, the numbers in Tables 2 and 3 with the respective scenario assessment allow this conclusion. As mentioned in section 3, the standard time period for a scenario analysis is considered to be 5 to 10 years. Insofar this assignment has to be regarded as preliminary. In addition, a major factor for the reduced numbers might have been caused by changing the publishing house in 2018 and not from this transformation.

## Discussion

This paper presented a methodology for evaluating the transformation of a SJ into an OAJ. The scenario analysis was described in general terms and with the specific facts for the use case of a journal transformation. Thus, there is now a list of suitable parameters and the description of five scenarios to monitor and evaluate a transformation from a SJ to an OAJ. This methodical approach was successfully tested with the journal MIM, and the current status and scenario for MIM were presented.

The application of the metric to evaluate the transformation of a journal from a SJ to an OAJ should be possible for a journal which is comparable to the MIM. The metric was developed to work for this use case for different journals. Especially the primary parameters should be applicable to any scientific journal. The level of detail of the parameters is left to the publishing house or editor of the journal. In addition, the metric is designed to be flexible enough to allow for an easy extension, for example, the possible final scenarios. It is also easy to add to or rearrange the parameters to evaluate the scenarios. Thus, the metric is adaptable to most of the contingencies known in scientific journals. This makes it also possible to use the metric in case changes are necessary.

The result that the MIM is on the way to a worst-case scenario is to be seen as positive after the current runtime of the transformation, because the runtime observations show that further measures such as intensive advertising measures or the registration in different open-access journal directories are necessary to successfully complete the transformation. In particular, the information about the current development is very valuable for the editor and the publisher to control the whole process.

## Limitations

During the Trans-O-MIM project and the preparation of this paper, various limitations arose, which limit the results and thus, the informative value of the facts presented.

Deviations in metrics in relation to other scientific journals are possible since other publishers may have different processes and thus different influences. However, extensive literature studies on the current status of Open Access across different disciplines have not provided any further indications of possible deviations from our metrics [2]. Therefore, we assume that the metrics can indeed be transferred to other scientific journals.

The metric was tested only on the example of MIM since no other SJ is known to have carried out a transformation into an OAJ using the tandem model. With the example of MIM it could be shown that the metric is an objective tool. Insofar it is possible to accompany and evaluate the transformation of a SJ into an OAJ and to take measures according to the resulting situations and numbers, which make it possible to achieve the desired target scenario.

There are various restrictions on the parameters, which are based on how much effort is required to collect the relevant data. This results in an imbalance between the effort required to collect data and the significance of the evaluation of the transformation. Among the primary parameters, cost parameters must be considered under this aspect. The costs for a journal are complicated to record since they are not available as individual costs for a journal and always refer to a mixed calculation based on all costs incurred. It is almost impossible to separate individual elements from this calculation, since determining the proportional costs is extremely time-consuming. In addition, costs are very company-specific and are often subject to secrecy. For the secondary parameters, it has been shown that the effort required to collect data for the parameter groups quality of publications, author satisfaction, demand comparison between the user groups, and topics between both journal parts is so high that the question of the actual benefit for an evaluation of the transformation arises. The evaluation can already be very well accompanied and evaluated based on collection and analysis of the primary parameters. Insofar a survey of the secondary parameters is not overly necessary, and it should be seen as additional information to make it possible to assess the scientific character and acceptance of the journal in the scientific community over several years. These parameters can thus contribute, for example, to sharpening the target group or investigating measures taken about scientific influence.

Our results for the MIM are only interim results related to the duration of the transformation and the investigation and monitoring with help from a scenario analysis. From these intermediate results, it can be seen that after the initial positive development, the transformation is now moving in the direction of an undesired final state. Being aware of this effect is positive because the editor and publisher can now take measures to counteract its development.

The analyzed data set includes only the original papers and the Focus Theme original papers. All other contributions such as editorials, discussions or memorandums at the invitation of the editor were not included in the analysis. We have therefore excluded 79 papers from the data set (January 2014 to September 2019). The main reason for this decision was that these papers do not undergo the classic review process and thus lack the quality control of papers by independent experts. In addition, many of these articles could not provide the necessary data for the planned evaluations.

A serious limitation that occurred during the project is the sale of Schattauer to Thieme. First of all, we have to mention that, fortunately, the Trans-O-MIM project received the same full support from Thieme as it has received from Schattauer before. This change was not foreseeable in the run-up to the transformation but had consequences for the transformation process. Work processes that were defined and developed in advance could, of course, not be reconciled with the new structures. For example, it was no longer possible to address the income and cost parameters of the journal within the framework of evaluation, since the collection of income and expenditure for the MIM journal was, for obvious reasons, not comparable and feasible between Schattauer and Thieme.

## Conclusion

The methodology of scenario analysis is suitable for evaluating the transformation from a SJ to an OAJ. The results of the thesis show that it is possible to use the parameter submissions, publications, acceptance rate, and duration to evaluate the transformation of a journal. The use of the other parameters should be decided depending on the cost-benefit ratio for the data collection. These parameters are well suited for assessing the scientific reach and the presence of the journal among the target groups. The interim status for the evaluation of the transformation of the MIM journal could be shown, and the developed scenarios are applied here. Corresponding to the situations described by the scenarios, measures should be taken within the framework of such a transformation to achieve the desired target scenario.

## Electronic supplementary material

ESM 1 (XLSX 74 kb)

## Data Availability

Data published as electronic supplementary material.

## Abbreviations

APC: Article Publication Charges
DFG: German Research Foundation
MIM: Methods of Information in Medicine
OA: Open Access
OAJ: Open Access-based Journal
PSP: Publication Service Providers
Schattauer: Schattauer GmbH
SJ: Subscription-based Journal
Trans-O-MIM: ‘Strategies, models and evaluation metrics for the goal-oriented, stepwise, sustainable and fair transformation of established subscription-based scientific journals into open-access-based journals with Methods of Information in Medicine as example’
Thieme: Georg Thieme Verlag KG
